# Reduced expression but not deficiency of *GFI1* causes a fatal myeloproliferative disease in mice

**DOI:** 10.1038/s41375-018-0166-1

**Published:** 2018-06-20

**Authors:** Jennifer Fraszczak, Charles Vadnais, Marissa Rashkovan, Julie Ross, Hugues Beauchemin, Riyan Chen, Damien Grapton, Cyrus Khandanpour, Tarik Möröy

**Affiliations:** 1 Institut de recherches cliniques de Montréal (IRCM), Montréal, QC Canada; 2 Département de microbiologie, infectiologie et immunologie, Université de Montréal, Montréal, QC Canada; 3 Division of Experimental Medicine, McGill University, Montreal, QC Canada; 4 Department of Hematology, University Hospital, Essen, Germany; 5 Department of Medicine A, Hematology, Oncology and Pneumology, University Hospital Münster, Münster, Germany

**Keywords:** Apoptosis, Myeloproliferative disease

## Abstract

Growth factor independent 1 (Gfi1) controls myeloid differentiation by regulating gene expression and limits the activation of p53 by facilitating its de-methylation at Lysine 372. In human myeloid leukemia, low *GFI1* levels correlate with an inferior prognosis. Here, we show that knockdown (KD) of Gfi1 in mice causes a fatal myeloproliferative disease (MPN) that could progress to leukemia after additional mutations. Both KO and KD mice accumulate myeloid cells that show signs of metabolic stress and high levels of reactive oxygen species. However, only KO cells have elevated levels of Lysine 372 methylated p53. This suggests that in contrast to absence of *GFI1*, KD of *GFI1* leads to the accumulation of myeloid cells because sufficient amount of *GFI1* is present to impede p53-mediated cell death, leading to a fatal MPN. The combination of myeloid accumulation and the ability to counteract p53 activity under metabolic stress could explain the role of reduced GF1 expression in human myeloid leukemia.

## Introduction

Growth factor independent 1 (Gfi1) is a transcription factor [1–7], which can repress target gene transcription by recruiting histone deacetylases, histone methyltransferases or histone de-methylases [3, 4, 8–14]. More recently, it has been suggested that Gfi1 binds to p53 [15] and forms a tripartite complex with LSD1. In this complex, Gfi1 recruits LSD1 to p53 and de-methylates its lysine 372 [16–19] limiting the ability of p53 to induce cell death [15]. As a consequence, Gfi1-deficient cells have more active p53 and are highly sensitive to apoptosis.

Gfi1 is mostly known for its key role in hematopoiesis [2, 20, 21], in particular in early lymphoid and myeloid development [22–26] and in hematopoietic stem cells [27–30]. It has been shown that absence of Gfi1 in mice or disabling mutations in the human *GFI1* gene leads to neutropenia and accumulation of monocyte and monocytic precursors [31–35]. Despite this accumulation of myeloid cells, Gfi1 deficiency alone, does not lead to the development of a myeloproliferative disease (MPN) or of an overt leukemia. Other events such as the overexpression of Bcl-2 [36] or a mutated and activated form of Kras are required to provoke an MPN like disease that can progress to acute myeloid leukemia (AML) in the absence of Gfi1 [37, 38].

Interestingly, low levels of Gfi1 have been associated with a worse outcome of both chronic myeloid leukemia [39, 40] and AML resulting from a myelodysplastic syndrome (MDS) [41, 42]. To study the relation between Gfi1 expression levels and myeloid leukemia, we have generated humanized *GFI1* “knock in” expressing the Human *GFI1* gene at WT levels (called “KI” mice) [38, 43] and mice expressing only a reduced level of *GFI1* called “KD” [26, 41]. KI and KD mice have been used to demonstrate that AML development is accelerated when Gfi1 expression is reduced [41]. However, the exact mechanism by which reduced Gfi1 expression levels accelerate or induce myeloid leukemia remains unclear and poorly understood.

Here, we show that low levels of Gfi1 alone can spontaneously cause a fatal, highly penetrant MPN predisposing to AML after accumulation of secondary mutations. Mice with a reduced expression of *GFI1* present the same myeloid differentiation defect as mice completely lacking Gfi1. However, myeloid cells from KD mice have a lower p53 activity leading to a better survival. Moreover, we present evidence that Gfi1 KO and KD cells show higher levels of reactive oxygen species and oxygen consumption. Our data not only indicate that low Gfi1 expression accelerates AML development and predisposes to very severe MPN, but also link Gfi1 to the regulation of genes controlling metabolisms.

## Experimental procedures

### Mouse strains

Gfi1 KO, *GFI1* KI, Gfi1 KD mice used in this study, have been previously described [26, 38, 41]. Trp53 KO mice were purchased from Jackson laboratory. Mice have been bred on to C57BL/6 genetic background for at least ten generations and were maintained in a Specific-Pathogen-Free Plus environment at the Institut de recherches cliniques de Montreal (IRCM). The Institutional Review Board of the IRCM approved all animal protocols and experimental procedures were performed in compliance with IRCM and CCAC (Canadian Council of Animal Care) guidelines.

### RNA-Seq profiling

RNA-Seq libraries were prepared using the Illumina TruSeq Stranded mRNA Kit according to the manufacturer’s instructions, and sequenced using the TruSeq PE Clusterkit v3-cBot-HS on an Illumina HiSEq 2000 system. Reads were aligned to the mm10 genome using Tophat v2.0.10 [44], processed with Samtools [45] and mapped to Ensembl genes using HTSeq. Differential expression was tested using the DESeq R package (R Coding Team). Raw data and processed reads are available on the Gene Expression Omnibus under the accession number GSE102957.

### Gene set enrichment analysis

The enrichment of selected biological functions was analyzed using the gene set enrichment analysis (GSEA) tool. Read counts for Ensembl genes from HTSeq were used and enrichment calculated using 1000 Gene Set permutations [46].

### Statistical analysis

Two tailed student’s *t*-test was used to calculate *p*-values where indicated. A *p*-value ≤0.05 was considered as statistically significant. Survival curves were analyzed by log-rank Mantel-Cox test using GraphPad Prism (GraphPad Software, La Jolla, CA, USA).

### Data availability

Raw data and processed reads from RNA-seq that support the findings of this study are available on the Gene Expression Omnibus under the accession number GSE102957.

## Results

### Mice expressing a low dose of Gfi1 develop fatal myeloproliferative disease

**Fig. 1:**
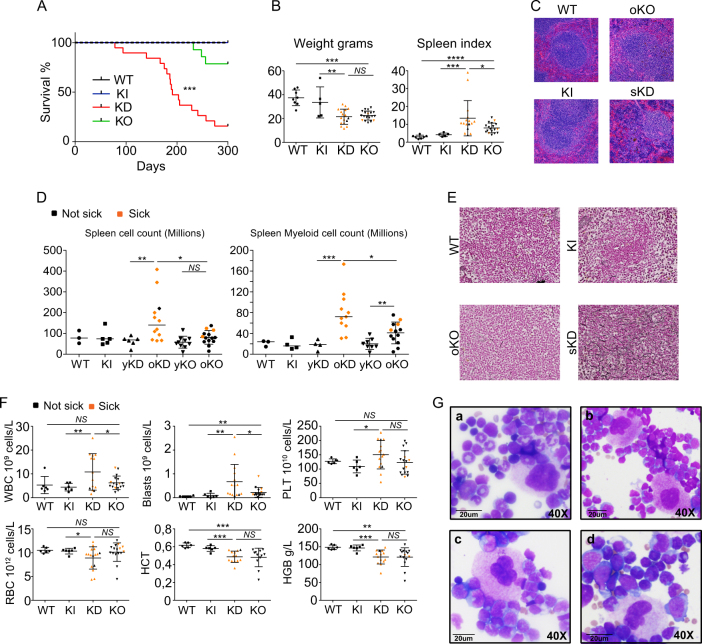
Mice expressing a low dose of Gfi1 develop a fatal myeloproliferative disease. **A** Survival curves of WT, KI, KD, and KO mice. Mice were considered sick when they showed symptoms such as weight loss, shaking, and impaired mobility. **B** Weight (left) and spleen index (right) of sick KD mice compared to age-matched WT, KI, and KO mice. **C** HE staining of spleens from WT, KI, sick KD (sKD), and age-matched old KO (oKO). **D** Total cell counts of splenic cells and myeloid cell counts in spleen of WT, KI, young KD (yKD), old KD (oKD), young KO (yKO), and old KO (oKO). Orange spots mean sick mice. **E** Reticulum staining of spleens from WT, KI, sick KD (sKD) and age-matched old KO (oKO). **F** White blood cell (WBC), red blood cell (RBC), platelets (PLT), hemoglobin levels (HGB), hematocrit (HCT), and blast counts in sick KD and age-matched WT, KI, and KO mice. **G** Examples of BM cytospins from control old KI mice (a), old KD mice (b), old KD mice starting to be sick (c), and sick old KD mice (d) after MGG staining. Magnification 40 ×.

Gfi1 KD mice (aged 4–12 weeks) showed the same myeloid differentiation arrest that has been previously described for Gfi1 KO mice namely an accumulation of Mac-1^+^Gr-1^low^ monocytes in bone marrow and spleen (Supplemental Fig. 1A–E). However, over an observation period of 300 days, almost all Gfi1 KD mice died with symptoms such as weight loss and splenomegaly, whereas almost all Gfi1 KO mice and all WT or KI animals survived this observation period (Fig. 1**A**-**C**). Spleen cellularity and total splenic myeloid cell counts including all Mac-1^+^ and Gr-1^+^ cells (Supplemental Fig. 1F) were increased in sick KD mice (Fig. 1**D**). In addition, sick KD animals also showed myelofibrosis, higher numbers of white blood cells, platelets, and blast cells but no anemia and higher serum levels of TNF-α compared to KO mice and controls (Fig. 1**E**, **F**; Supplemental Fig. 1G), indicating ongoing inflammation. Moreover, analysis of BM cytospins from KD mice (Fig. 1**G**) showed an increase of megakaryocytes with a normal morphology and an accumulation of immature myeloid precursors but no signs of dysplasia in any lineages. This suggests that KD mice succumb to a fatal MPN very similar to the essential thrombocythemia type where an elevated platelet count is observed in patients (Fig. 1**F**). Supporting this hypothesis, we found that KD had higher numbers of myeloid related progenitors such as MPP3 and GMP cells (Supplemental Fig. 1H), but did not show any changes in HSC numbers (data not shown). Moreover, KD and KO showed a tendency for more erythroid and megakaryocyte progenitors (Supplemental Fig. 1I, J) possibly to compensate the decrease of red blood cells and support the higher level of platelets observed in the blood, respectively.

To test the aggressiveness of this disease, we transplanted BM cells from three different sick KD mice into two lethally irradiated recipient mice each. Four out of six animals that received BM cells from sick KD mice succumbed to a disease with similar symptoms as the original KD mice (Supplemental Fig. 1K–M). However, these mice did not develop the disease faster than KD mice themselves, which is indicative for a MPN, but not for leukemia.

### Low Gfi1 expression predisposes to myeloid leukemia in the presence of additional mutational events

**Fig. 2:**
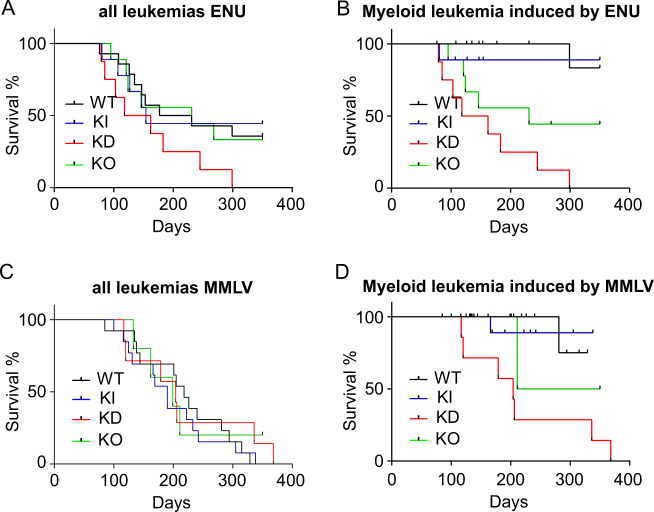
Low level of GFI1 predisposes to myeloid leukemia. **A** and **C** Survival curves of mice. WT, KI, KD, and KO mice were injected with ENU 3 to 4 weeks after birth (**A**) or with MMLV 24 to 72 h after birth (**C**). Mice were analyzed when they developed disease symptoms. **B** and **D** Survival curves of mice developing myeloid leukemia. WT, KI, KD, and KO mice were injected with ENU 3 to 4 weeks after the birth (**B**) or MMLV 24 to 72 h after the birth (**D**). Mice were analyzed when they developed disease symptoms.

It is known that MPN patients can progress to AML over time if additional mutations occur. We investigated whether this situation can be mimicked in our mouse model and used the carcinogen ethylnitrosourea (ENU) or the non-acute retrovirus Moloney murine leukemia virus (MMLV), known to cause lymphoid or myeloid leukemia [1, 47–49]. After injections of ENU or MMLV, we found that KD mice died with the same latency compared to controls (Fig. 2**A**, **C**) but not from the same disease. Indeed, the majority of control WT and KI mice developed T-cell leukemia (T-ALL) after injection of ENU or MMLV (Supplemental Fig. 2A–F). Surprisingly, none of the KD mice injected either with ENU or MMLV developed T-ALL, but succumbed to myeloid leukemia with high penetrance (Fig. 2**B**, **D**, and Supplemental Fig. 2G–J). The injected KO mice developed myeloid leukemia with a lower incidence compared to KD mice and some KO mice also developed T-ALL but with a delay as was previously shown [15]. These data suggest that reduced expression levels of *GFI1* can predispose to the development of a AML in the presence of additional genetic events or mutations (Supplemental Fig. 2K).

### Restoration of normal Gfi1 expression levels abrogates the MPN and myeloid leukemia predisposition in KD animals

**Fig. 3:**
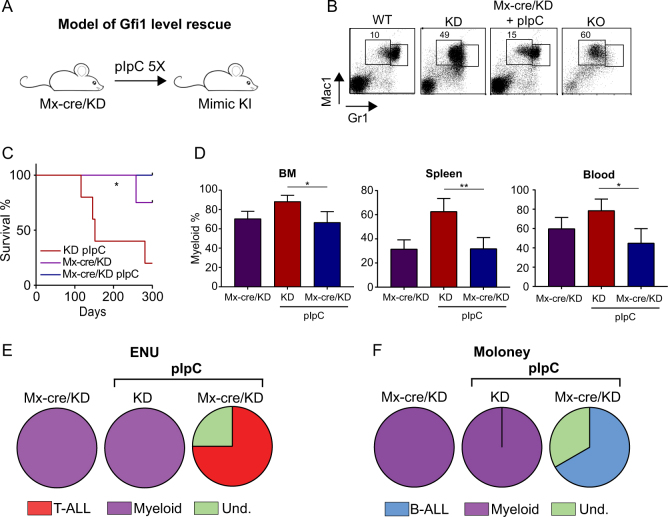
Gfi1 level rescue abrogates the myeloproliferative disease and myeloid leukemia predisposition. **A** Mouse model of Gfi1 level rescue. Mx-cre/KD mice injected with five doses of pIpC every 2 days have a deletion of the Neo cassette in the Human GFI1 transgene rescuing the level of Gfi1 (KI). **B** Representative FACS plot of BM cells from Mx-cre/KD mice injected with pIpC, WT, KD, and KO mice. BM cells were stained with Mac1 and Gr1 antibodies and analyzed by flow cytometry. **C** Survival curves of Mx-cre/KD mice injected or not with pIpC and KD mice injected with pIpC. Mice were considered sick when they showed symptoms such as weight loss, shaking, and less mobility. **D** Percentages of myeloid cells in sick KD mice injected with pIpC and Mx-cre/KD mice injected or not with pIpC. BM, blood, and splenic cells from sick mice were stained for the expression of myeloid markers Gr1 and Mac1 and analyzed by flow cytometry. **E** and **F** Proportions of T-ALL, myeloid leukemia, B-ALL, and undeterminate disease in sick Mx-cre/KD mice receiving or not pIpC and sick KD mice receiving also pIpC, 80 days after injections with ENU (**E**) or MMLV (**F**).

To demonstrate that a reduced level of *GFI1* is responsible for the observed disease in KD mice, we set-up an experiment in which full, wild-type Gfi1 expression is restored in these animals. Such as restoration can be achieved in KD mice by expressing Cre recombinase to excise the floxed neo cassette [43]. Hence, we generated Mx-Cre/Gfi1KD mice and injected them with pIpC to activate Cre expression, which led to increased Gfi1 expression levels, similar to the level found in KI thymocytes (Fig. 3**A** and Supplemental Fig. 3A). However, we also found that the Mx-Cre/KD mice, which did not receive any pIpC had a slightly increased level of Gfi1 suggesting background activity of the Cre recombinase. Most significantly, Mx-Cre KD mice that had received pIpC no longer showed the myeloid differentiation arrest seen in KD mice and in KO animals (Fig. 3**B**). In addition, they no longer developed a fatal MPN (Fig. 3**C**, **D**). Most of the Mx-Cre Gfi1-KD transgenic mice also remained disease free although no pIpC was injected, probably due to “leaky” expression of the Cre recombinase.

Interestingly, we also found that Mx-Cre KD mice injected with MMLV and ENU and later with pIpC developed predominantly lymphoid leukemia (Fig. 3**E**, **F**) while mice expressing a low level of Gfi1 still showed the myeloid accumulation leading to myeloid leukemia (Supplemental Fig. 3B, C). These results indicate that restoration of normal levels of Gfi1 expression abrogates the predisposition of Gfi1 KD mice to develop a fatal MPN and AML upon ENU or MMLV injection confirming the significance of Gfi1 levels in both diseases.

### Reduced *GFI1* expression confers increased plating and reconstitution efficiency

**Fig. 4:**
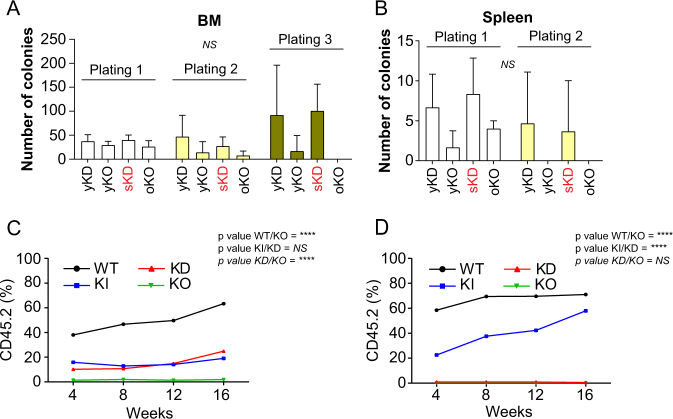
Sick KD mice showed increase myeloid progenitors in the BM and in the spleen. **A** and **B** Colony assay performed with 5000 BM cells (**A**) or 5000 splenic cells (**B**) from young KD (yKD) and KO (yKO), sick KD (sKD) and age-matched KO (oKO) mice. Every week, cells were harvested and plated in 1 mL of methylcellulose. **C** An equivalent number of CD45.1^+^ BM carrier cells and CD45.2^+^ BM cells from WT, KI, KD, and KO mice were transplanted into lethally irradiated recipient CD45.1 mice. After, 4, 8, 12, and 16 weeks, blood from transplanted recipient mice was analyzed for the expression of CD45.1 and CD45.2 by FACS. **D** A serial secondary transplantation assay was performed with BM cells from the 1st round transplanted mice. After, 4, 8, 12, and 16 weeks, blood from transplanted mice was analyzed for the expression of CD45.1 and CD45.2 by FACS.

To further investigate why KD and KO myeloid cells show this different behavior, we performed colony assays. We observed that cells from BM and spleen of both young, healthy KD mice and old, sick KD animals generated higher number of colonies than cells from age-matched KO mice or controls after a second or third re-plating (Fig. 4**A**, **B**, and Supplemental Fig. 4A). However, colony formation of KD cells was limited to three rounds of plating. Competitive transplantation assays with BM cells from WT, KI, KD, and KO mice showed a better reconstitution capacity for KD than for KO BM cells (Fig. 4**C**). Transplanted KD and KI BM cells reached higher percentages of CD45.2^+^ cells in the blood but never equivalent to WT BM cells (Fig. 4**C**). Cells from transplanted mice were then used for a secondary transplantation experiment where we found that CD45.2^+^ cells from both KD and KO cells were lost (Fig. 4**D**). These experiments suggested that cells from KD animals do not have a better self-renewal capacity than cells from WT or KI mice.

To test whether the difference in reconstitution between KD, KI, and WT cells can be explained by a lower homing capacity, we transplanted BM cells form WT, KI, KD, and KO mice with or without competitors into CD45.1 recipients and tested the presence of CD45.2^+^ cells after 24 h. No difference in homing was observed (Supplemental Fig. 4B, C). However, the frequency of progenitor cells (lin^−^) among the CD45.2^+^ population was significantly lower in mice transplanted with KD and KI cells compared to WT transplants (Supplemental Fig. 4D). These data suggest that the number of progenitor cells migrating into the BM of recipient mice after transplantation is different, potentially explaining the delay of the reconstitution (Fig. 4**C**). We also found that the engraftment capacity after 6 weeks, was similar between WT, KI, and KD but was decreased for KO cells (Supplemental Fig. 4E). This decrease is also seen in a competition context 16 weeks after transplantation where we could not find any KO cells (Supplemental Fig. 4F–H). We hypothesized that the improved plating efficiency and enhanced initial reconstitution capacity of KD cells could be due to their greater resistance to cell death compared to KO cells.

### Cells from KD mice showed a better survival and resistance to stress compared to KO cells

**Fig. 5:**
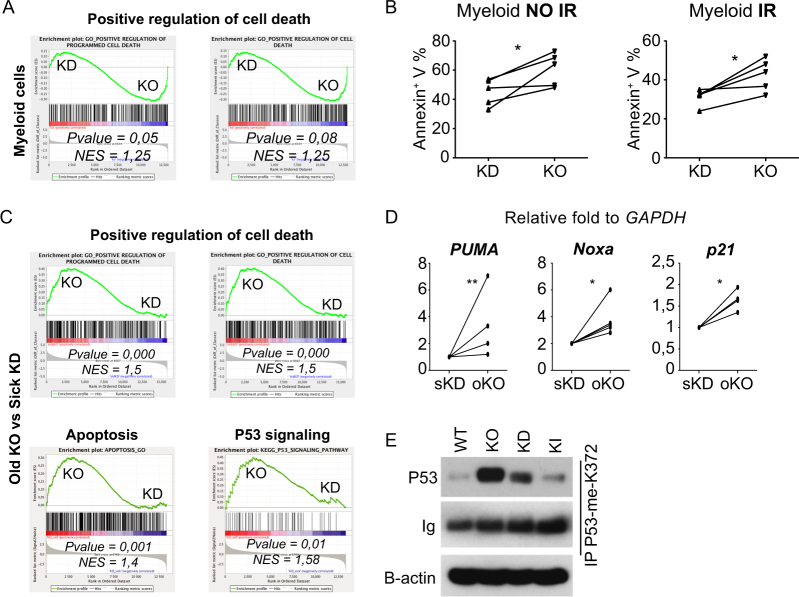
KD mice showed a different survival and p53 responses compared to KO mice. **A** RNA-seq analysis was performed to compare sorted myeloid cells from young and not sick KD and age-matched KO. Example of Gene Set Enrichment Analysis of significantly modulated functions: positive regulation of cell death. NES = normalized enrichment score. **B** Percentages of annexin V positive cells in sorted KD and KO myeloid cells from healthy and young mice after or not irradiation (5 Gy). **C** RNA-seq analysis was performed to compare BM cells from sick KD and age-matched KO (“old KO”). Gene Set Enrichment Analysis of positive regulation of cell death, apoptosis, and p53 signaling. NES = normalized enrichment score. **D** *Puma, Noxa*, and *p21* expression in old KO BM cells compared to sick KD BM cells. All values were normalized to the expression of the *Gapdh* gene and are presented relative to cDNA from age-matched sick KD BM cells. **E** Methyl-p53 expression (lysine 372) measured by western blot in WT, KI, KD, and KO BM cells.

To test this, we compared RNA-seq expression profiles from young KD and KO BM cells, but did not find a significant enrichment of gene signatures related to apoptosis or p53 signaling nor did we find differences between KD and KO BM cells in apoptosis (Supplemental Fig. 5A, B). However, after a stress signal (γ-irradiation), we observed a better survival of BM cells, which correlated with a lack of upregulation of p53 effector genes such as Puma, Noxa, or p21 in BM KD cells vs. the equivalent BM KO cells (Supplemental Fig. 5C, D). Considering that most of the cells found in KD and KO BM are myeloid cells, we sorted this population based on Gr1 and Mac1 expression (Supplemental Fig. 5E) and analyzed them by RNA-Seq. We found that KO myeloid cells show a higher enrichment for gene sets involved in positive regulation of cell death compared to KD myeloid cells (Fig. 5**A**). This enrichment correlated with a higher capacity to undergo apoptosis in KO myeloid cells regardless of whether a stress signal was present (Fig. 5**B**).

Next, we compared RNA expression profiles from whole BM cells from sick KD mice and age-matched (old) KO animals and observed an enrichment of gene sets for positive regulation of cell death, apoptosis and p53 signaling (Fig. 5**C**) in old KO cells over sick KD cells. This was in agreement with an upregulation of Puma, Noxa, and p21 in old KO cells compared to sick KD cells (Fig. 5**D**). This indicated that Gfi1 KD cells lack the previously reported sensitivity of Gfi1 KO cells [15, 36] to react with increased apoptosis in response to stress signals. This data suggested also that p53 activity is differently regulated in KD cells than in KO cells. Indeed, we found that KO BM cells had a higher level of p53 methylated on lysine 372, known to be associated with an active p53, compared to KD BM cells (Fig. 5**E**).

**Fig. 6:**
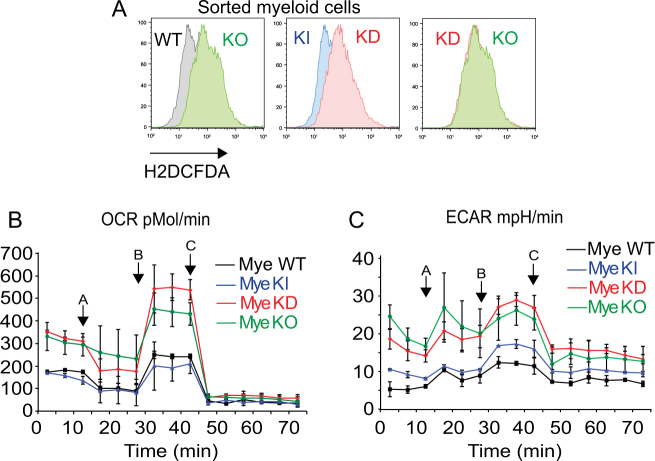
KD and KO cells showed a metabolic deregulation. **A** Representative histogram showing ROS production by sorted myeloid cells from WT, KI, KD, and KO mice. BM cells were stained for Gr1 and Mac1 and sorted based on an identical gate and analyzed for ROS production by staining with H2DCFDA. **B** and **C** Oxygen consumption rate (OCR) (**B**) and extracellular acidification rate (ECAR) (**C**) by sorted myeloid cells from WT, KI, KD, and KO measured with the seahorse technology by using the Mito Stress Cell Kit. A = oligomycin, B = FCCP, and C = rotenone.

To explore which events could lead to a basal activation of p53, especially in myeloid cells, we measured the production of reactive oxygen species (ROS) by myeloid cells. We found an increased production of ROS in KO and KD sorted myeloid cells compared to control cells WT and KI (Fig. 6**A**). We also found a slight, but variable tendency of sick KD myeloid cells to produce more ROS than old KO myeloid cells (Supplemental Fig. 5F), associated to an enrichment of sets of genes involved in oxidative phosphorylation (Supplemental Fig. 5G).

This data suggested a possible deregulation of the metabolic activity in KD and KO myeloid cells causing an increased ROS production. To test this hypothesis, we used the Seahorse analyzer and found that sorted myeloid cells from young KD and KO mice had similarly elevated mitochondrial oxygen consumption and acidification rates when compared to WT and KI cells (Fig. 6**B**, **C**). We also found a small increase in maximal oxygen consumption and acidification in total BM cells from sick KD mice compared to age-matched KO mice (Supplemental Fig. 5H). This indicated that the myeloid population in KD and KO mice has a higher metabolic activity and possibly as a consequence of this, higher ROS levels than control cells. This again could be a trigger for further progression of myeloid malignancies, since ROS has been implicated in myeloid pathogenesis and prognosis [50–52].

### KO mice missing one or two alleles of p53 develop a fatal MPN similar to KD animals

**Fig. 7:**
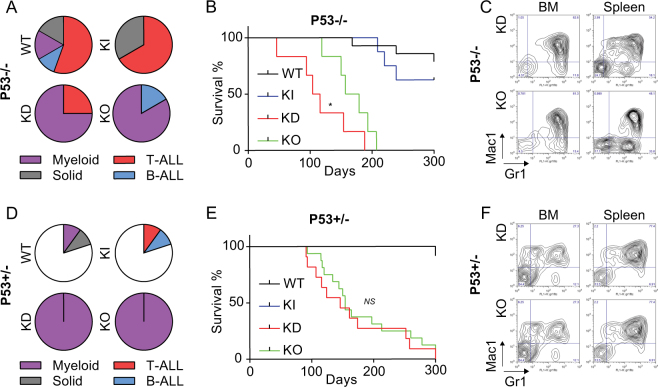
KD mice showed a different p53 signaling profile compare to the KO mice. **A** Proportions of T-ALL, myeloid leukemia, B-ALL, and solid tumors in sick WT p53-/-, KI p53-/-, KD p53-/-, and KO p53 -/- mice. Mice were considered sick when they showed symptoms such as weight loss, shaking, and less mobility. **B** Survival curves of sick WT p53-/-, KI p53-/-, KD p53-/-, and KO p53-/- mice developing a myeloid disease. **C** Representative FACS plots of sick KD p53-/- and KO p53-/- developing a myeloid disease. **D** Proportions of T-ALL, myeloid leukemia, B-ALL, solid tumors, or no disease in sick WT p53 + /-, KI p53 + /-, KD p53 + /-, and KO p53 + /- mice. Mice were considered sick when they showed symptoms such as weight loss, shaking, and less mobility. **E** Survival curves of sick WT p53 + /-, KI p53 + /-, KD p53 + /-, and KO p53 + /- mice developing a myeloid disease. **F** Representative FACS plots of sick KD p53 + /- and KO p53 + /- developing a myeloid disease

Given the lower methylation of p53 at K372 associated with a lower activity of p53 in KD over KO cells, it is possible that KD mice are less capable to eliminate accumulated myeloid cells than KO mice. If this hypothesis was true, decreasing or eliminating p53 activity in KO mice should lead to the same MPN that is observed in KD mice. To test this, we deleted one or both alleles of p53 in Gfi1 KO and KD mice as well as in WT and KI control animals. As previously reported, WT and KI, p53^−/−^ mice developed predominantly T-ALL. However, KD/p53^−/−^ and KO/p53^−/−^ animals succumbed mainly to MPN with a 100% penetrance (Fig. 7**A**–**C**). In addition, while control WT and KI/p53^+/−^ mice remained free of disease, KO/p53^+/−^ and KD/p53^+/−^ mice developed MPN with similar latencies (Fig. 7**D**–**F**). We confirmed that deletion of one allele of p53 and thus a decreased p53 expression (Supplemental Fig. 6A) abrogated the difference of *Puma* expression between sick KD/p53^−/−^, KD/p53^+/−^, KO/p53^−/−^, and KO/p53^+/−^ BM cells (Supplemental Fig. 6B, C). Moreover, KO and KD/p53^+/−^ BM cells showed very similar colony plating efficiencies and bone marrow reconstitution capacities (Supplemental Fig. 6D–F). This indicated that KD and KO cells differ in their regulation of p53 activity and when this difference is eliminated or reduced, both KD and KO mice are predisposed to develop a fatal MPN.

## Discussion

**Fig. 8:**
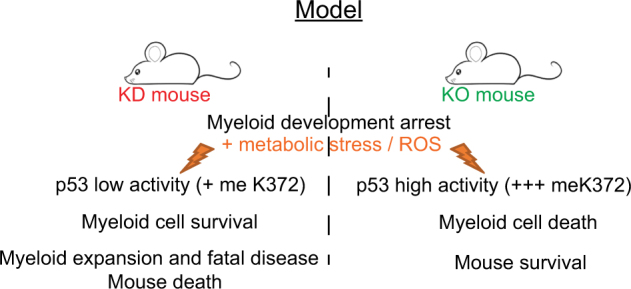
Model comparing KD and KO mice. In this model, KD and KO mice have a myeloid arrest associated with ROS production, which can activate p53 signaling. KO mice show a higher p53 activity leading to the myeloid cell death and the survival of the mice. In contrary, mice expressing a low level of GFI1 can contain p53 activity by demethylating p53 lysine 372 supporting the survival and the expansion of the abnormal myeloid cells causing the mouse death.

We show here that a low dose of Gfi1 leads to a fatal MPN with high penetrance in mice, which can progress to AML in the presence of additional mutations. We present evidence that this MPN arises as a consequence of a combination of three effects caused by low Gfi1 expression (Fig. 8): a myeloid differentiation arrest with accumulation of myeloid cells; altered metabolic activity leading to elevated ROS levels and reduced ability to activate a p53 cell death pathway upon stress signals.

### Gfi1 knockdown, but not knockout, causes a fatal MPN that can progress to myeloid leukemia

Several symptoms observed in Gfi1 KD mice observed over a period of almost 1 year [53–55] suggest that a MPN develops in these animals [56, 57]. Moreover, our finding that reduced Gfi1 expression levels almost exclusively orients the effect of both mutagenic agents, ENU and MMLV [1, 47–49], towards a myeloid leukemia is additional evidence that a low Gfi1 dose predisposes for MPN or AML. Since it is well known that patients with MPN can develop AML after secondary mutations [58–61], it is conceivable that our mouse models mimic the situation in human AML patients who already carry additional mutations and in combination with low *GFI1* expression have a faster disease progression from a previous less malignant MPN.

Our KD mouse model was engineered in such a way that a restoration of wild-type levels is possible. Thus, a rescue to normal Gfi1 expression leads to an elimination of MPN in mice, which clearly demonstrated that MPN is a direct consequence of reduced *GFI1* expression. This was confirmed by additional reports, showing that enforced expression of *GFI1* leads to maturation of myeloid leukemic cells in vitro and in vivo [43]. These findings also suggested that *GFI1* expression levels could be actionable for leukemia therapy. For instance, complete elimination of *GFI1* expression would render cells sensitive to p53-mediated cell death or restoration of reduced to normal *GFI1* expression could lead to differentiation of myeloid cells and delay or eliminate progression to leukemia. Drugs that initiate myeloid differentiation are already used in therapy to treat promyelocytic leukemia and a restoration of reduced *GFI1* expression to normal levels would have a similar and potentially therapeutic effect [43].

### Gfi1 dose has an effect on the regulation of p53 activity and the survival of myeloid cells

We have previously shown that Gfi1 can recruit LSD1 to p53 to remove methyl groups from lysine 372 [15, 16]. In the absence of Gfi1, p53 is, therefore, highly methylated at this residue and active [15] and accelerates programmed cell death in hematopoietic cells upon a stress signal [15, 62]. One of the major differences between KD and KO cells is that this high propensity of p53-mediated cell death is only observed in KO cells, very likely because the K372 methylation is much higher in KO cells as in KD cells. Elimination of one or two alleles of p53 eliminates this difference between KD and KO and as a result now KD and KO mice develop the same fatal MPN. This supports the notion that the regulation of p53 activity depends on the dose of Gfi1. This experiment also provides strong evidence for the hypothesis that a fatal MPN occurs in KD mice and not in KO mice because of a differential regulation of p53-mediated cell death.

### Gfi1 can regulate metabolic activity in myeloid cells and metabolic stress

One of the new discoveries of this study is the link between Gfi1 and the regulation of metabolic activity and stress such as the level of ROS. Reduced or absence of *GFI1* in young and healthy mice cause a higher oxygen consumption and acidification. For Gfi1-deficient cells to initiate an accelerated p53-mediated apoptosis, a stress signal is required since cells at steady state from KD or KO mice do not show different cell death rates. One explanation for such a stress signal could be the higher metabolic activity in cells, which most likely also causes the higher ROS level [63, 64]. Since a higher metabolic activity and ROS levels are also found in young and healthy KO and KD cells compared to controls, it is possible that this is not a consequence of ageing, but a direct result of aberrant gene regulation.

It is thus likely that KO mice respond to the higher level of ROS by activating p53 in myeloid cells and eliminating them by apoptosis so no MPN can develop. In contrast, KD mice cannot eliminate their myeloid cells by activating p53, since the low level of K372 methylation limits this step leading to myeloid accumulation over time and MPN in ageing animals (Fig. 8). In support of this, previous reports have shown that ROS can support the MPN development in humans [65–69] and can even be involved in chronic leukemia [70, 71]. According to these observations, using ROS inhibitors could perhaps reduce the aggressiveness of the disease in KD mice but would probably not fully rescue the phenotype of immature myeloid cell accumulation, which is provoked by the deregulations of specific myeloid gene sets. Hence it is conceivable that in KD mice, high levels of ROS could increase the severity of the MPN with a high potential to progress to a more malignant state.

## Electronic supplementary material

SUPPLEMENTAL INFORMATION

Supplemental Figure 1

Supplemental Figure 2

Supplemental Figure 3

Supplemental Figure 4

Supplemental Figure 5

Supplemental Figure 6
