# Supplementary material for: Reduced expression but not deficiency of GFI1 causes a fatal myeloproliferative disease in mice
Source: Leukemia. 2018 Jun 20;33(1):110–21. doi: 10.1038/s41375-018-0166-1 (PMC6326955; doi:10.1038/s41375-018-0166-1)
Supplement: Supplementary file 1 — SUPPLEMENTAL INFORMATION [file 41375_2018_166_MOESM1_ESM.docx]

**SUPPLEMENTAL INFORMATION**

**SUPPLEMENTAL EXPERIMENTAL PROCEDURE**

**Flow cytometry analysis**

BM, spleen, blood and thymus from mice were analyzed by flow cytometry for CD4, CD8, CD3, CD44, CD25, Gr1, Mac1, B220 and CD19 expression markers. For progenitor analysis lineage cocktail (B220, CD3, CD4, CD8, Gr1, Mac1, Ter119, NK1.1, DX5, CD5, IgM), c-Kit, Sca1, Flt3, CD150 and CD48 antibodies were used. CD45.1 and CD45.2 were used for the competitive assay analysis. Antibodies directly conjugated to a fluorochrome or biotin, were purchased from BD Biosciences, eBiosciences or Biolegend. Cells were analyzed with a LSR I (BD Biosciences) and FlowJo software.

**Tumorigenesis assays**

3-4-week-old mice were injected intraperitoneally with N-ethyl-N-nitrosourea (ENU) or newborn mice were injected intraperitoneally with Moloney Murine Leukemia Virus (MMLV). Animals were sacrificed by CO_2_ euthanasia upon moribund appearance. Then, BM, splenic, blood and thymic cells were analyzed by flow cytometry to determine the leukemia type. Blood cell count was also measured with an Advia Hematology system.

**Rescue of Gfi1**

In the GFI1-KD mice, the targeted locus includes a floxed neo cassette oriented in anitisence to the endogenous Gfi1 transcription. The presence of the Neo cassette therefore leads to a low expression of the Human GFI1 “knockin” cDNA. KD mice were crossed with Mx-Cre transgenic mice to generate a model, in which the neo cassette can be removed and the normal GFI1 expression can be restored. To activate Cre-mediated recombination, Mx-cre KD mice were injected intraperitoneally with 250 ul of a 2mg/ml solution of pIpC in PBS, every second day, for five injections.

**Transplantation assay**

*Non competitive transplantation*: 5x10^6^ bone marrow cells (CD45.2) from sick KD mice or control KI mice were transplanted into lethally irradiated (9.5 Gy) CD45.1 mice. Animals were sacrificed by CO_2_ euthanasia upon moribund appearance.

*BM competitive and serial transplantations*: 5x10^5^ or 1x10^6^ bone marrow cells (CD45.2) from WT, KI, KD and KO mice were transplanted with an equal number of CD45.1 bone marrow cells (Ratio 1:1) into lethally irradiated (9.5 Gy) CD45.1 recipient mice. At 4, 8, 12 and 16 weeks, blood from transplanted mice was analysed for the expression of CD45.1 and CD45.2 markers. After 16 weeks, the animals were sacrificed and 3x10^6^ BM cells were transplanted a second time into lethally irradiated (9.5 Gy) CD45.1 recipient mice. BM cells and splenic cells were also analyzed to detect the presence of CD45.2^+^ progenitor cells, CD45.2^+^ myeloid cells or CD45.2^+^ lymphoid cells by flow cytometry.

**Homing assay**

*Non-competitive homing experiment*: 1x10^6^ bone marrow cells (CD45.2) from WT, KI, KD or KO mice were transplanted into lethally irradiated (9.5 Gy) CD45.1 mice. BM and splenic cells from recipient mice were analyzed 20-24 hours after transplantation for CD45.2 and CD45.1 expression and analyzed by flow cytometry.

*Competitive homing experiment*: 1x10^6^ bone marrow cells (CD45.2) from WT, KI, KD or KO mice were transplanted with an equal number of CD45.1 bone marrow cells (Ratio 1:1) into lethally irradiated (9.5 Gy) CD45.1 recipient mice. Then BM and splenic cells from recipient mice were analyzed like previously described in non-competitive homing experiment.

**RNA isolation and real-time PCR.**

For RNA isolation, cells were lysed in Trizol (Life technology) or in RLT buffer with 10% of β-mercaptoethanol (RNeasy Micro kit, Qiagen). RT-PCR was performed using Superscript II (Invitrogen). Real time PCR was performed in triplicates on the ViiA7 Real time PCR machine (Life technologies) in SYBR Green Master mix (Applied Biosystems). The expression of the gene of interest was calculated relative to the *Gapdh* mRNA (ΔC_T_) and is presented as “fold induction” relative to values obtained with the respective control (set as “1-fold”).

**Western blot analysis and Co-immunoprecipitation.**

After isolation, cells were lysed in NP-40 buffer in the presence of protease inhibitor cocktail (Complete Mini, Roche Diagnostics). The following antibodies were used for immunoblotting and co-immunoprecipitation: anti lamin B (C-20, Santa Cruz), anti Gfi1 (R&D systems), anti β-actin (Ac-15, Sigma-Aldrich), anti p53 (C-20, Cell signaling) and anti-methyl-p53 (K372, Abcam).

**Colony assay**

5000 BM or splenic cells were plated into 1 mL methylcellulose (Methocult M3434, Stemcell Technologies), cultured on 35 mm dishes. After 7 days of culture, images of the entire surface were collected using a 5X objective of a Zeiss Axiovert S100TV microscope and analyzed by MATLAB. Colonies were harvested and re-plated following the same procedure into methylcellulose every week for serial re-plating until exhaustion.

**Cell death**

AnnexinV staining was performed using the AnnexinV-APC and AnnexinV detection kit I (BD biosciences) by following the manufacturer’s instructions. Cells were analyzed with LSR I (BD Biosciences).

**ROS staining**

BM cells or sorted myeloid cells were incubated at 37 °C for 5 min with 5-(and-6)-carboxy-2′,7′-dichlorodihydrofluorescein diacetate (carboxy-H2DCFDA; Invitrogen, 1 μM) in pre-warmed RPMI medium, washed twice in pre-warmed medium and analyzed by FACS.

**Oxygen rate consumption analysis**

BM cells or sorted myeloid cells were cultured in assay media (XF base medium (Agilent), 200 mg glucose, 0,5 mM sodium pyruvate and 2 mM glutamine, pH 7.4) for 1 h without CO_2_ prior to measurement of O_2_ consumption by XF^e^24 (Seahorse Bioscience) with sequential addition of 1 μM of oligomycin, 1 μM of FCCP and 0,5 μM of rotenone.

**SUPPLEMENTAL FIGURE LEGENDS**

**Supplemental Figure 1: Mice expressing a low dose of Gfi1 develop a fatal myeloproliferative disease**

(**A**) FACS analysis of BM for myeloid cells. BM cells from healthy and young (4-12 weeks) WT, KI, KD and KO mice were stained for Gr1 and Mac1.

(**B**) Cytospins with BM cells from healthy and young (4-12 weeks) WT, KI, KD and KO mice. Cells were centrifuged on a glass slide and stained with May-Grünwald-Giemsa solution.

(**C**) Absolute numbers of monocytes in BM from healthy and young (4-12 weeks) WT, KI, KD and KO mice.

(**D**) FACS analysis of spleen for myeloid cells. Splenic cells from healthy and young (4-12 weeks) WT, KI, KD and KO mice were stained for Gr1 and Mac1.

(**E**) Absolute numbers of monocytes in spleen from healthy and young (4-12 weeks) WT, KI, KD and KO mice.

(**F**) Gating in red of myeloid cells (including Mac1^+^, Gr1^+^ and Mac1^+^Gr1^+^) in WT, KI, sick KD and KO mice used for the analysis in figure 1D.

(**G**) IL-6 and TNF-α concentrations in serum from WT, KI, young KD (yKD), young KO (yKO), old KD (oKD) and old KO (oKO).

(**H**) Cell numbers of MPP3 (Lin^-^Sca1^+^cKit^+^CD150^-^CD48^+^) and GMP (Lin^-^Sca1^-^cKit^+^CD34^low^CD16/32^+^) in the BM from WT, KI, KD and KO of young (y) and old (o) mice. Orange triangle means sick mouse.

(**I**) Number of proerythroblasts (CD71^+^ and ter119^low^) and ter119^+^ cells in the BM from WT, KI, KD and KO mice.

(**J**) Number of megakaryocyte progenitors (MkP: Lin^-^cKit^+^Sca1^-^CD42d^low^CD41^low^CD16/32^+^CD150^+^CD9^+^) and mature megakaryocytes (mat. Mk: Lin^-^cKit^+^Sca1^-^CD42d^+^CD41^+^) in the BM of WT, KI, KD and KO mice.

(**K**) Survival curves of mice transplanted with BM cells from sick KD mice and control KI mice. 5 million BM cells from sick KD and control KI mice were transplanted into lethally irradiated CD45.1 recipient mice. Mice were considered sick when they showed symptoms such as weight loss, shaking, and low mobility.

(**L**) FACS analysis of BM, splenic and blood cells from sick CD45.1 mice transplanted with sick KD mice BM cells. Cells were stained for CD45.1. CD45.2, Gr1 and Mac1.

(**M**) Cytospins with BM cells from sick CD45.1 mice transplanted with sick KD mice BM cells. Cells were centrifuged on a glass slide and stained with May-Grünwald-Giemsa solution.

**Supplemental Figure 2: Low level of GFI1 predisposes to myeloid leukemia**

(**A and D**) Survival curves of mice developing T cell leukemia and lymphoma after ENU (**A**) or MMLV (**D**) injections. WT, KI, KD, KO mice were injected with either ENU 3 to 4 weeks after the birth or with MMLV 24 to 72 hours after the birth. Mice were analyzed when they developed disease symptoms.

(**B and E**) Representative FACS plots of sick mice. BM and splenic cells from sick mice injected with either ENU (**B**) or MMLV (**E**) were stained for the expression of T cell markers CD4 and CD8 and analyzed by flow cytometry.

(**C and F**) White blood cell (WBC) number in sick WT, KI, KD and KO mice developing a T-ALL after either ENU (**C**) or MMLV (**F**) injection.

(**G and I**) Representative FACS plots of sick KD and KO mice. BM and splenic cells from sick mice injected with either ENU (**G**) or MMLV (**I**) were stained for the expression of myeloid markers Gr1 and Mac1 and analyzed by flow cytometry.

(**H and** **J**) White blood cell number (WBC) and large unstained cells (LUC, blasts) percentages in sick WT, KI, KD and KO mice developing a myeloid leukemia after ENU (**H**) or MMLV (**J**) injections.

(**K**) Proportions of T-ALL, myeloid leukemia, B-ALL, solid tumor and undeterminate disease in sick WT, KI, KD and KO mice injected either with ENU (left) or MMLV (right).

**Supplemental Figure 3: Gfi1 level rescue abrogates the myeloproliferative disease and myeloid leukemia predisposition**

**(A**) GFI1 expression by western blot in the thymus from Mx-cre/KD mice injected or not with pIpC, KD mice and KI mice. Lamin B was used as a loading control. Low = Low exposure and High = High exposure.

(**B**) Representative FACS plots of sick KD mice treated with pIpC and Mx-cre/KD mice treated or not with pIpC 80 days after ENU injections. BM and splenic cells from sick mice were stained for the expression of myeloid markers Gr1 and Mac1 and analyzed by flow cytometry.

(**C**) Representative FACS plots of sick KD mice treated with pIpC and Mx-cre/KD mice treated or not with pIpC 80 days after MMLV injections. BM and splenic cells from sick mice were stained for the expression of myeloid markers Gr1 and Mac1 and analyzed by flow cytometry.

**Supplemental Figure 4: Sick KD mice showed increase myeloid progenitors in the BM and in the spleen**

**(A**) Colony assay performed with 5000 BM cells from young (y) and old (o ; s = sick) WT, KI, KD and KO BM cells. BM cells were plated in 1 mL of methylcellulose and counted every 7 days. Every week, cells were harvested and plated in 1 mL of methylcellulose. A maximum of 3 successive platings were performed for this experiment.

(**B**) 1x10^6^ bone marrow cells (CD45.2) from WT, KI, KD or KO mice were transplanted with an equal number of CD45.1 bone marrow cells (Ratio 1:1) into lethally irradiated (9.5 Gy) CD45.1 recipient mice. Then BM and splenic cells from recipient mice were analyzed for CD45.1 and CD45.2 expression by flow cytometry, 20-24 hours after transplantation.

(**C**), (**D**) and (**E**) Bone marrow cells (CD45.2) from WT, KI, KD or KO mice were transplanted into lethally irradiated (9.5 Gy) CD45.1 recipient mice. 20-24 hours, BM and splenic cells from recipient mice were analyzed for CD45.2 expression (**C**) and for CD45.2 lineage negative cells in the BM (**D**). 6 weeks after transplantation, CD45.2^+^ cell numbers were measured in the spleen and in the BM as well as the number of LSK cells in the BM (**E**).

(**F), (G**) and (**H**) An equivalent number of CD45.1^+^ BM carrier cells and CD45.2^+^ BM cells from WT, KI, KD and KO mice were transplanted into lethally irradiated recipient CD45.1 mice. (**F**) After 16 weeks, transplanted mice were analyzed for the presence of CD45.2^+^ cells in the spleen and BM. BM cells from recipient mice were also analyzed for progenitor cells 16 weeks after the transplantation. Number of LSK cells (**G**) and representative FACS for progenitors (lin^-^) expressing c-Kit, Sca1, CD150 and CD48 (**H**).

**Supplemental Figure 5: KD mice showed a different survival and p53 responses compared to KO mice**

**(A**) RNAseq analysis performed to compare BM cells from young and “healthy” KD (“young KD”) and age matched young KO (“young KO”). Gene Set Enrichment Analysis of apoptosis and p53 signalings. NES: normalized enrichment score.

(**B**) Percentage of annexin V positive cells in KD and KO total BM cells.

(**C**) Percentage of annexin V positive cells in KD and KO total BM cells after irradiation (**IR**, 5Gy).

(**D**) *Puma, Noxa and p21* expression in BM cells from WT, KI, KD and KO mice after or not irradiation (5Gy). All values were normalized to the expression of the *Gapdh* gene and are presented relative to cDNA from no irradiated WT BM cells.

(**E**) Representative FACS gate for myeloid cell sorting from KD and KO mice for GSEA analysis.

(**F**) Two representative FACS plots from two independent experiments of ROS staining in sick KD (sKD) and age-matched old KO (oKO) BM cells. BM cells were stained with the H2DCFDA specific ROS probe.

(**G**) RNAseq analysis performed to compare BM cells from sick KD and age matched KO (“old KO”). Example of Gene Set Enrichment Analysis of significantly modulated function: oxidative phosphorylation (OXPHOS). NES: normalized enrichment score.

(**H**) OCR and ECAR by BM cells from sick KD and age-matched KO mice (old KO) mice measured as previously in Figure 6B and C.

**Supplemental Figure 6: KD mice showed a different p53 signaling profile compare to the KO mice**

(**A**) Analysis of *p53* gene expression by RT PCR in KD (left, orange) and KO (right, yellow) mice missing or not one or two p53 alleles (total BM cells). All values were normalized to the expression of the *Gapdh* gene and are presented relative to cDNA from KD p53+/+ for the KD samples or KO p53+/+ for the KO samples.

(**B**) *Puma* gene expression by RT PCR in total BM cells from sick p53-/- KD and KO, and p53+/- KD and KO mice. All values were normalized to the expression of the *Gapdh* gene and are presented relative to cDNA from KD p53-/- or KD p53+/- BM cells.

(**C**) *Puma* gene expression by RT PCR in total BM cells from WT p53+/-, KI p53+/-, KD p53+/- and KO p53+/- mice after irradiation (5Gy).

(**D**) Colony assay performed with 5000 BM cells from WT, KI, KD and KO p53 +/- mice. BM cells were plated in 1 mL of methylcellulose and counted every 7 days. Every week, cells were harvested and plated in 1 mL of methylcellulose. A maximum of 3 successive platings were performed for this experiment.

(**E-F**) An equivalent number of CD45.1^+^ BM carrier cells and CD45.2^+^ BM cells from WT, KI, KD and KO p53 +/- mice were transplanted into lethally irradiated recipient CD45.1 mice. (**E**) After, 4, 8, 12 and 16 weeks, blood from transplanted recipient mice was analyzed for the expression of CD45.1 and CD45.2 by FACS. (**F**) After 16 weeks, transplanted mice were analyzed for the presence of CD45.2^+^ cells in the BM and spleen.
