# Supplementary figures and images for: Reduced expression but not deficiency of GFI1 causes a fatal myeloproliferative disease in mice
Source: Leukemia. 2018 Jun 20;33(1):110–21. doi: 10.1038/s41375-018-0166-1 (PMC6326955; doi:10.1038/s41375-018-0166-1)

Supplemental Figure 1

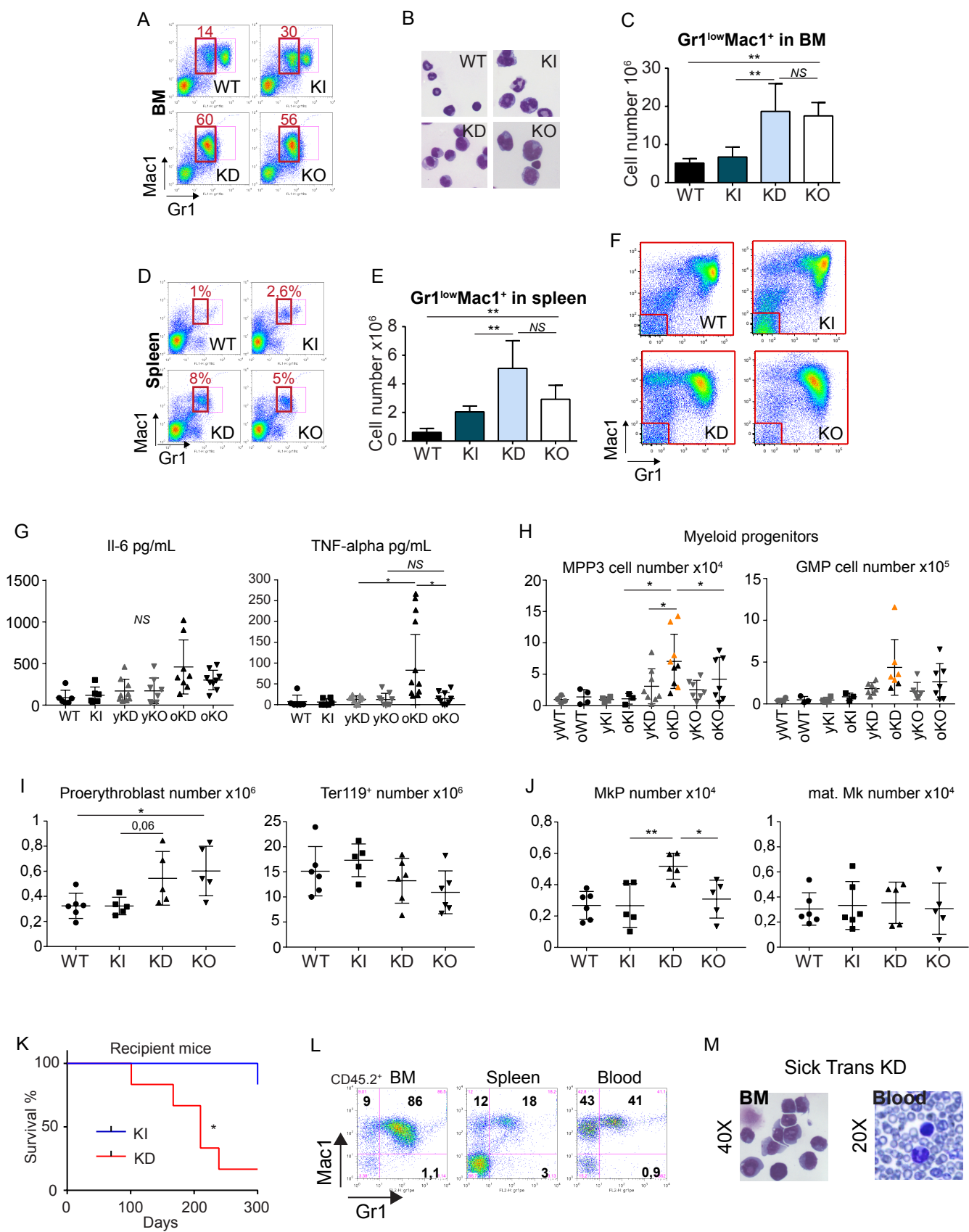

Supplement: Supplementary file 2 — Supplemental Figure 1 [file 41375_2018_166_MOESM2_ESM.pdf]

Supplemental Figure 2

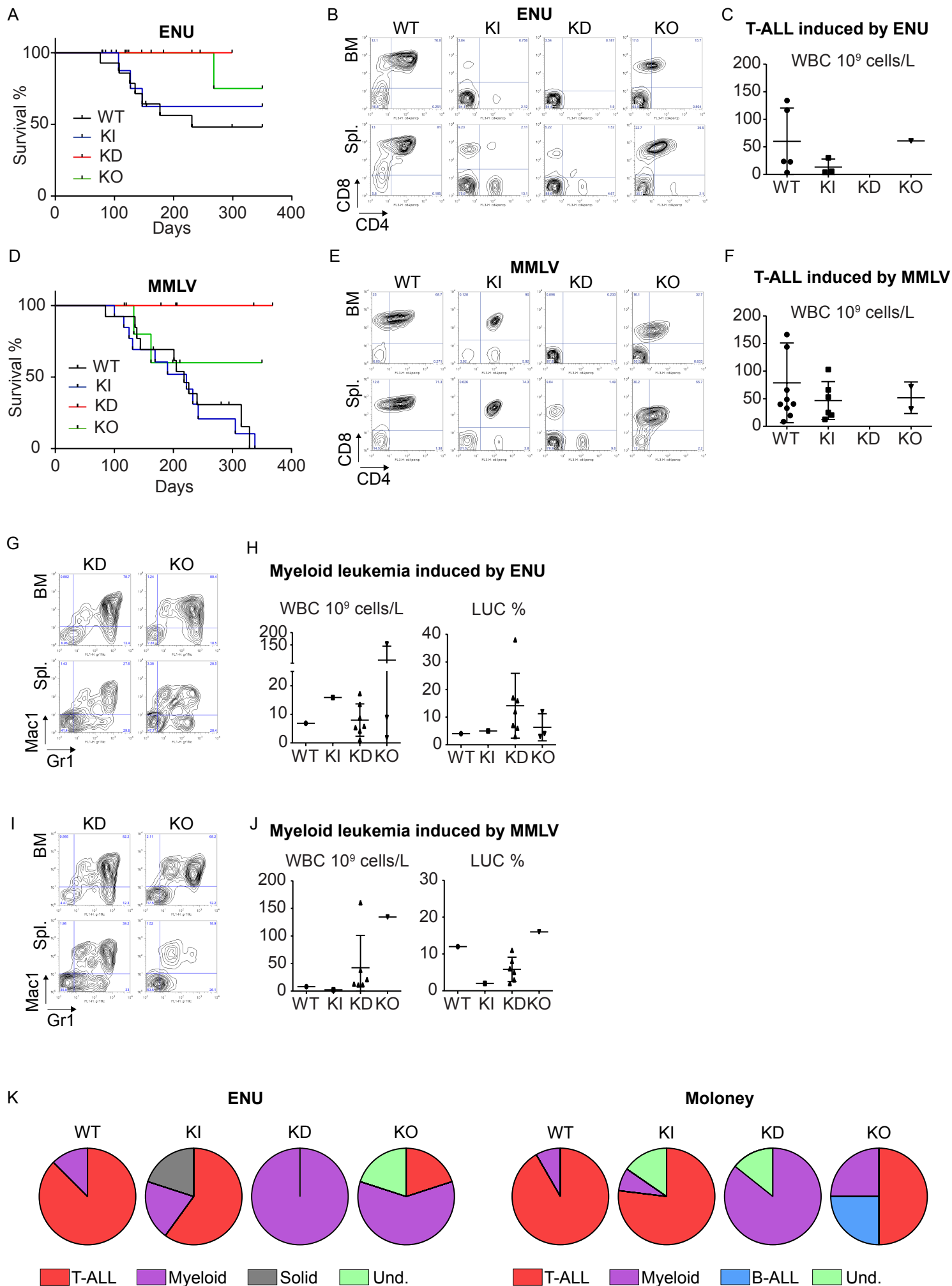

Supplement: Supplementary file 3 — Supplemental Figure 2 [file 41375_2018_166_MOESM3_ESM.pdf]

Supplemental Figure 3

A

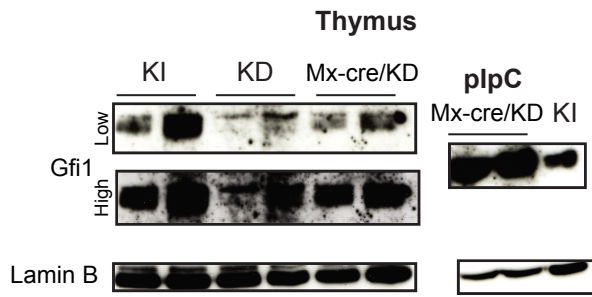

B

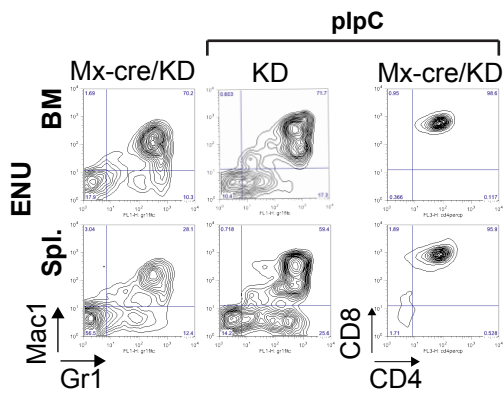

C

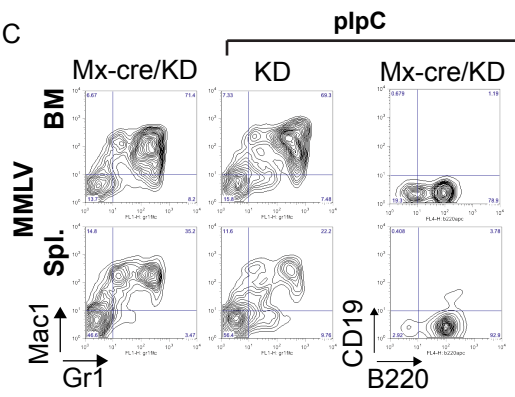

Supplement: Supplementary file 4 — Supplemental Figure 3 [file 41375_2018_166_MOESM4_ESM.pdf]

Supplemental Figure 4

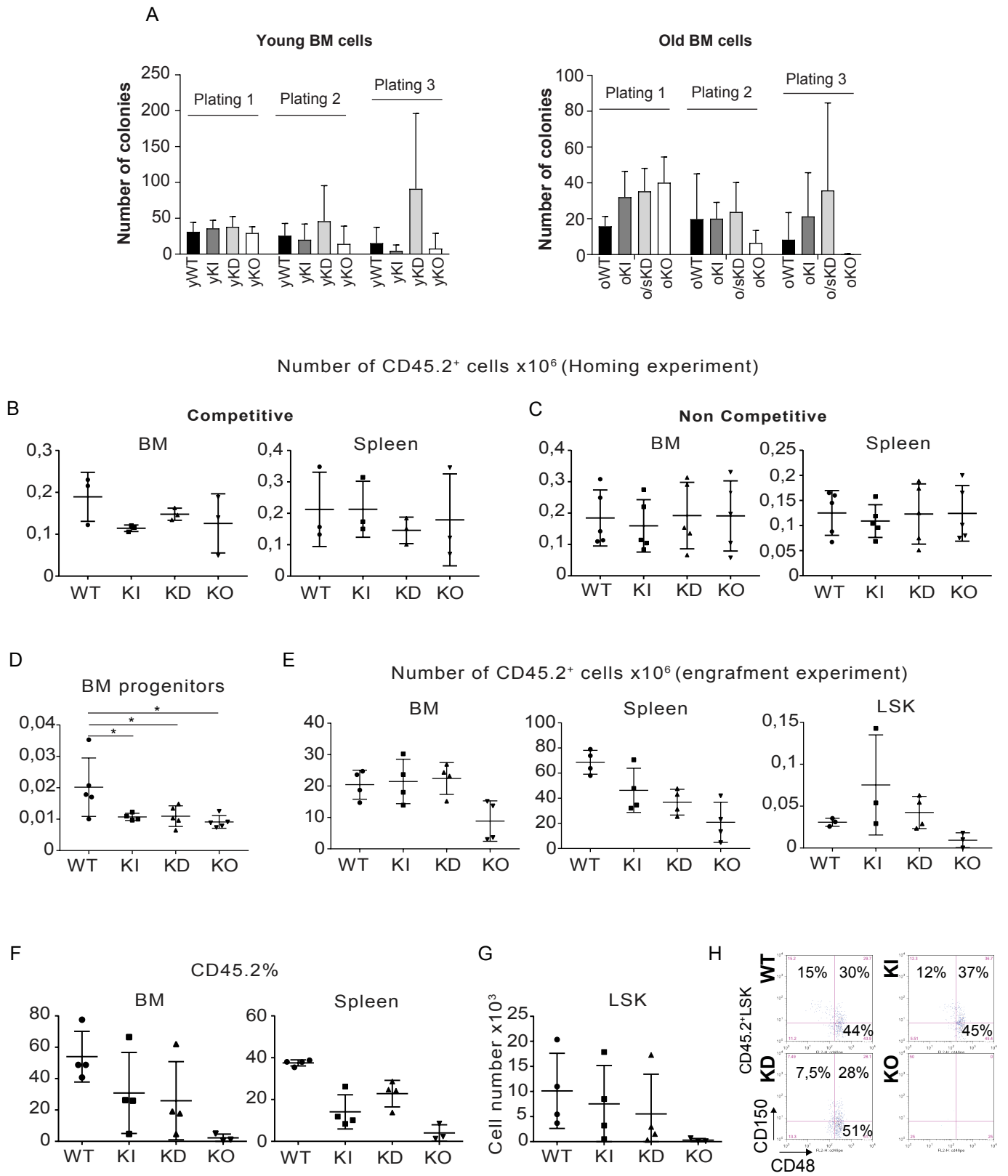

Supplement: Supplementary file 5 — Supplemental Figure 4 [file 41375_2018_166_MOESM5_ESM.pdf]

Supplemental Figure 5

A

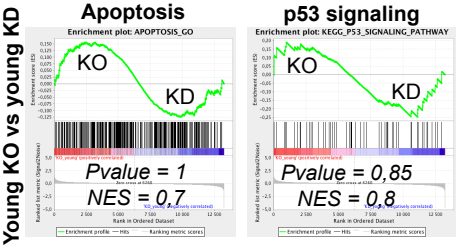

B

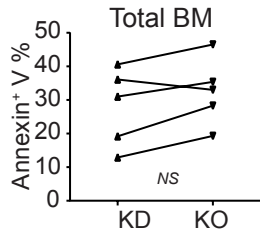

C

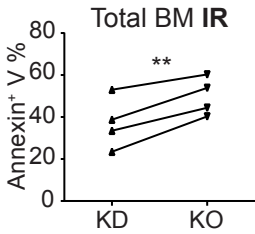

D

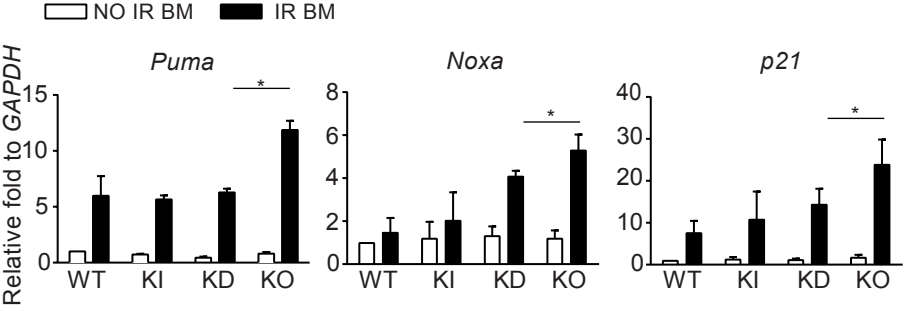

E

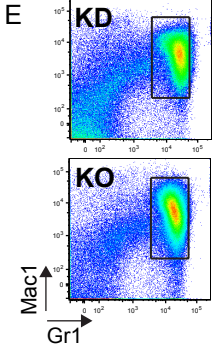

F

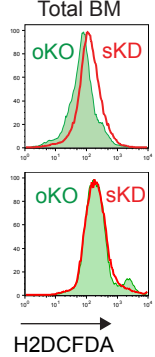

G

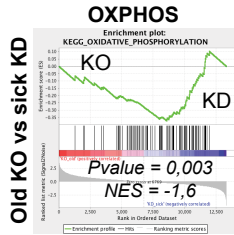

H

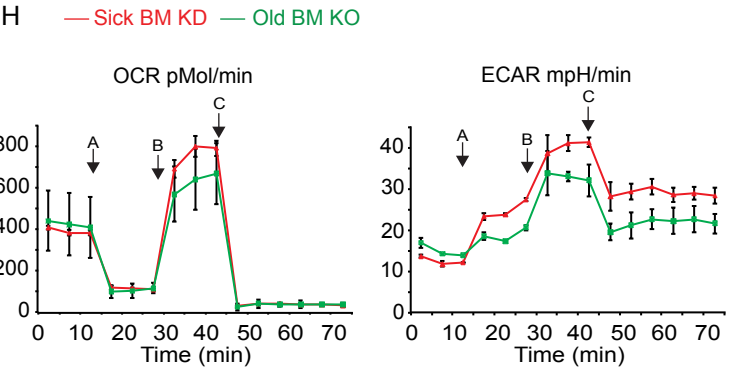

Supplement: Supplementary file 6 — Supplemental Figure 5 [file 41375_2018_166_MOESM6_ESM.pdf]

Supplemental Figure 6

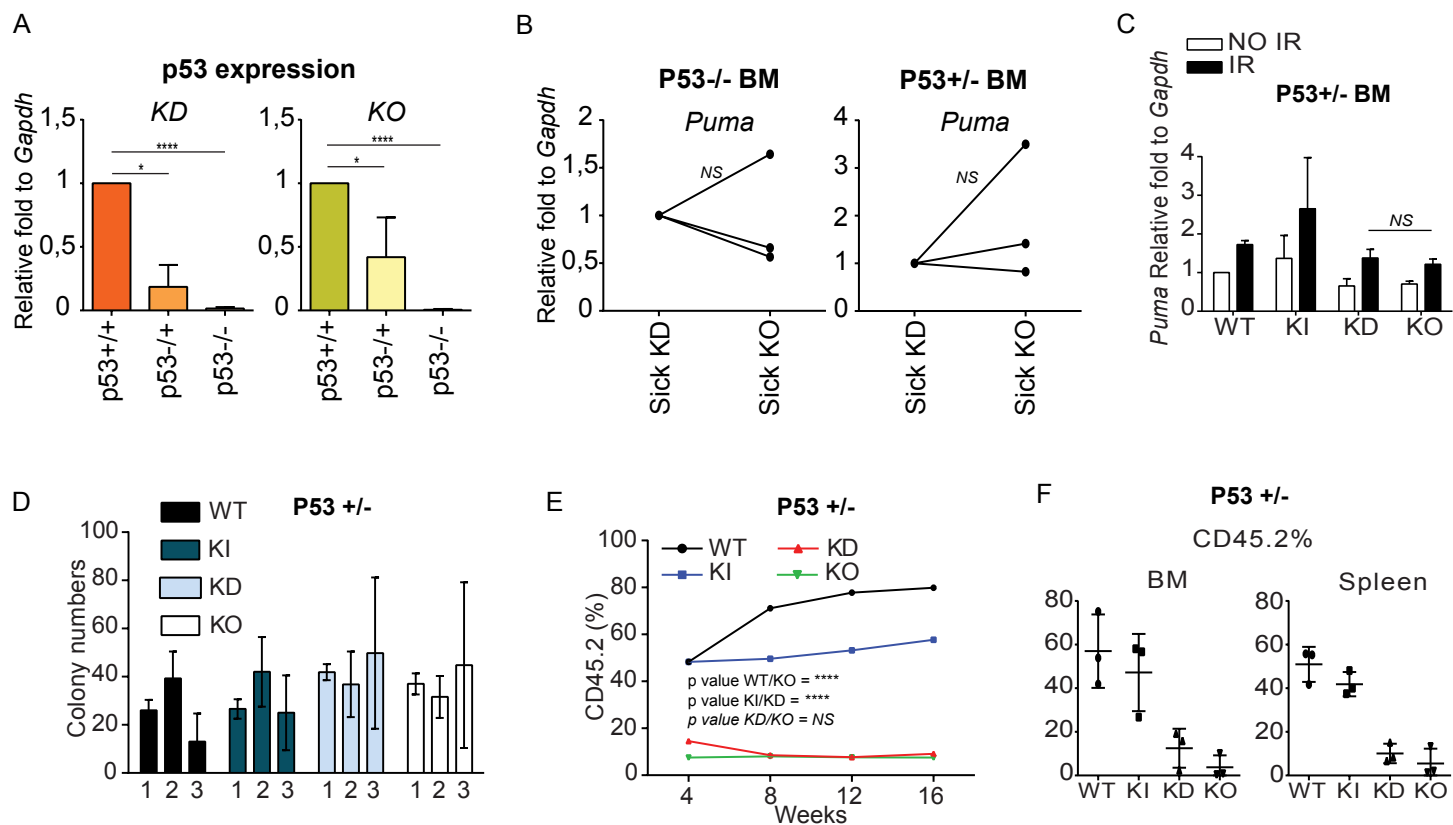

Supplement: Supplementary file 7 — Supplemental Figure 6 [file 41375_2018_166_MOESM7_ESM.pdf]
